# A Bayesian approach for temporally scaling climate for modeling ecological systems

**DOI:** 10.1002/ece3.2092

**Published:** 2016-03-28

**Authors:** Max Post van der Burg, Michael J. Anteau, Lisa A. McCauley, Mark T. Wiltermuth

**Affiliations:** ^1^ U.S. Geological SurveyNorthern Prairie Wildlife Research Center8711 37th StreetJamestownNorth Dakota58401; ^2^ U.S. Geological SurveyNorthern Prairie Wildlife Research CenterSouth Dakota State University8711 37th 5 St SEJamestownNorth Dakota58401; ^3^ Present address: A105 Russell LabsThe Nature Conservancy Center for Science and Public Policy 1510 EFort Lowell Rd TucsonAZ 85719

**Keywords:** Bayesian hierarchical models, drought indices, hydrology, semipermanent wetlands, Standardized Precipitation Evapotranspiration Index, Prairie Pothole Region

## Abstract

With climate change becoming more of concern, many ecologists are including climate variables in their system and statistical models. The Standardized Precipitation Evapotranspiration Index (SPEI) is a drought index that has potential advantages in modeling ecological response variables, including a flexible computation of the index over different timescales. However, little development has been made in terms of the choice of timescale for SPEI. We developed a Bayesian modeling approach for estimating the timescale for SPEI and demonstrated its use in modeling wetland hydrologic dynamics in two different eras (i.e., historical [pre‐1970] and contemporary [post‐2003]). Our goal was to determine whether differences in climate between the two eras could explain changes in the amount of water in wetlands. Our results showed that wetland water surface areas tended to be larger in wetter conditions, but also changed less in response to climate fluctuations in the contemporary era. We also found that the average timescale parameter was greater in the historical period, compared with the contemporary period. We were not able to determine whether this shift in timescale was due to a change in the timing of wet–dry periods or whether it was due to changes in the way wetlands responded to climate. Our results suggest that perhaps some interaction between climate and hydrologic response may be at work, and further analysis is needed to determine which has a stronger influence. Despite this, we suggest that our modeling approach enabled us to estimate the relevant timescale for SPEI and make inferences from those estimates. Likewise, our approach provides a mechanism for using prior information with future data to assess whether these patterns may continue over time. We suggest that ecologists consider using temporally scalable climate indices in conjunction with Bayesian analysis for assessing the role of climate in ecological systems.

## Introduction

With climate change becoming more of concern, many ecologists are including climate variables in their system and statistical models. Such efforts have included assessing how species may respond to changes in climate (e.g., Niemuth et al. 2008), as well as how landscape features that function as wildlife habitat, such as wetlands, might respond to wetting and drying periods (e.g., Johnson et al. 2005, 2010). For ecosystems, like wetlands, that appear to respond to patterns of both drought and deluge, linking some aspect of system function to climatic fluctuations is important for understanding habitat dynamics (Anteau 2012). Typically, these patterns might be inferred by relating a response variable to weather variables like temperature or precipitation (Forcey et al. 2011). Others have used indices that represent more complex relationships between climate variables (e.g., Thogmartin and McKann 2014). Indices, like the Palmer Drought Severity Index (PDSI; Palmer 1965), have an intuitive appeal in modeling responses to climate because they represent departures from average conditions, where positive values indicate relative wetness and negative values indicate relative dryness. The PDSI, is also useful because it relies on information about water balance (i.e., water inputs and evapotranspiration) to estimate those departures. This can allow for modeling responses to expected available moisture in a way that simply using temperature or precipitation data does not.

While PDSI is a popular choice for an index, it may be somewhat limited in its usefulness for those interested in modeling systems responding to finer spatiotemporal variability in climate. As others have pointed out, PDSI is computed using a fixed time interval and is often most useful for describing regional climate variability (Guttman 1998; Vicente‐Serrano et al. 2010). For modeling phenomena on a more local scale, some analytical flexibility with regard to computing a climate index would be useful. One index that seems to possess this flexibility is the Standardized Precipitation Index (SPI). As the name implies, SPI estimates departures from average precipitation over time (McKee et al. 1993). This index can be computed in both a spatially and temporally relevant way, but it may be limited by the fact that it does not model departures based on a water balance like the PDSI. This could be a problem because both temperature and precipitation interact to determine regional and local climate. As an improvement in drought modeling, Vicente‐Serrano et al. (2010) presented an alternative index called the Standardized Precipitation Evapotranspiration Index (SPEI). Their approach allows for estimation of a multiscalar index that captures departures from average conditions using precipitation and potential evapotranspiration over user‐defined timescales. However, choice of timescale seems to be left to expert knowledge or intuition. Perhaps a more objective solution could be found by treating the appropriate timescale as an unobservable parameter to be estimated.

We explored this idea of estimating the timescale for computing both SPI and SPEI with a case study focused on understanding how wetland hydrology responds to fluctuations and changes in climate. We based our analysis on the data presented in McCauley et al. (2015), who modeled wetland water surface area as a function of climate and land use variables. In their analysis, they used a model selection approach to find an appropriate timescale for SPEI, and then included that index in subsequent models containing other variables. The variable selection approach they used may be problematic because it does not allow the analyst to explore all of the potential parameter and variable combinations that are possible in a model. Additionally, one does not have a flexible way to assess whether the effect of climate may have changed over time. One could accomplish this by specifying many more model combinations with a different timescale parameter in each model. But this approach would be very cumbersome and would not necessarily allow one to assess dynamics in climate as a model parameter.

Bayesian hierarchical models provide an alternative approach that allows for the modeling of more complex relationships between parameters in a model (Clark 2007). Likewise, the Bayesian approach treats all parameters as random draws from distributions and thus focuses on characterizing those distributions, rather than simply generating point estimates. In this case study, we developed a hierarchical model for analyzing wetland water surface areas as a function of SPEI‐derived indices, where the temporal scaling parameter was a variable to be estimated. Much like the models of McCauley et al. (2015), we considered variability of surface areas between two eras, but we allowed the scaling parameter to vary between the two periods. Our intent with this case study was to estimate timescale parameters used in computing drought indices, determine whether those parameters were different between eras, and then draw inferences about whether the estimates could help to explain some of the variability we observed in wetland water surface areas.

## Materials and Methods

Our study was based on the work of McCauley et al. (2015), which was focused on modeling variability of hydroperiods, as indexed by water surface areas, in relatively closed‐basin wetlands called “potholes.” The wetlands in their study were located in the North Dakota, U.S.A., portion of the “Prairie Pothole Region” (PPR) of North America. The quality of these kinds of wetlands as habitat appears to be related to hydroperiod (e.g., Snodgrass et al. 2000), so any alterations of those hydroperiods are likely to have effects on wetland‐dependent wildlife populations. Natural variation in water levels is known to be caused by regional wetting and drying periods (Winter and Rosenberry 1998; Johnson et al. 2004). However, climate on a smaller scale is likely to be highly variable throughout the PPR, so modeling wetland responses to climate requires capturing finer spatial and temporal variability in climate.

### Study area and wetland selection

The procedure that McCauley et al. (2015) used for selecting sample wetlands can also be found in Anteau and Afton (2008). In general, they focused on two ecoregions within the larger PPR: Missouri River Coteau and Northern Glaciated Plains. Sampling clusters were allocated to each of the ecoregions depending on the size of the region. This resulted in 6 and 14 sampling clusters in each of the ecoregions, respectively. They then randomly selected three 94‐km${}^{2}$ townships within each cluster and randomly selected three wetlands from within each township. Our analysis was based on 147 wetlands ranging from 0.5 to 750 ha.

### Measuring wetland water surface area

McCauley et al. (2015) measured water surface areas in wetlands using historical aerial photographs. In short, they acquired photos of each wetland in all available years prior to 1970 from digital (USGS Earth Explorer and scanned photos from U.S. Fish and Wildlife Service) and print (U.S. Department of Agriculture, Natural Resources Conservation Service, and Farm Service Agency county offices) sources. McCauley et al. (2015) surmised that observed water surface areas in those images were less affected by widespread wetland consolidation drainage. They also obtained contemporary photographs from the USDA National Agriculture Imagery Program for the years spanning 2003–2010, except 2007 and 2008. Observed surface areas from this era were thought to be likely influenced by consolidation drainage (Anteau 2012; McCauley et al. 2015). They digitized all imagery and subsequent measurements of water surface areas were made using ArcGIS v.10 (ESRI 2015; McCauley et al. 2015). They also made numerous assumptions when computing surface areas. For example, when the water boundary was not easily seen because it was hidden by emergent vegetation, they interpolated the water area to the halfway point between the visible water line and the outer edge of the emergent vegetation. Additionally, when a road crossed a wetland, they digitized the boundary of the wetland at the road.

Because these assumptions had the potential to underestimate wetland size, we decided to model water surface area as the ratio of observable water surface area to basin area. In essence, this meant that we were modeling change in the relative area of water, rather than the absolute area. We used basin areas that were computed by Wiltermuth (2014). Stated simply, he defined wetland basins as topographic depressions that collect surface water and identified these depressions using high‐resolution digital elevation models (3 m pixel LiDAR or 5 m pixel InSAR). He then used contour areas at the spill point elevation for each basin to assign basin area values. As with water surface areas, he truncated the size of basins bisected by roads. This ensured that water surface areas for bisected wetlands could, in practice, only be as large as the associated basin. While this does not correct for the issues caused by truncating either area relative to the road, we contend that focusing on the ratio of water area to basin area (i.e., proportion area) potentially reduces some of the problems associated with skewed absolute water surface areas.

### Estimating drought index

We calculated drought indices for each wetland using temperature and precipitation estimates from the Parameter‐elevation Regressions on Independent Slopes Model (PRISM Climate Group, Oregon State University). These estimates were interpolated to a 2.5 arcmin grid using digital elevation (DEM) and linear regression models that relate weighted weather station observations to elevation (Daly et al. 2008). We downloaded monthly precipitation and minimum and maximum temperature values for 1895 to 2011 for each wetland. We used the SPEI as well as the SPI in our analysis. For the calculation of SPEI, we used the Hargreaves potential evapotranspiration equation (Hargreaves 1994), which uses precipitation and minimum and maximum temperature values. As mentioned above, both indices require the specification of a timescale for which the index should be computed. The choice of this time parameter is then used in an autocorrelation function to weight the amount of temporal aggregation used in computing the index. It is this amount of temporal aggregation, and the effect that has on predicting proportion area, that we were primarily interested in estimating for this study.

For our analysis, we assumed a uniform correlation function and considered timescales ranging from 1 to 120 months for SPEI and 1 to 3 months for SPI. We considered these monthly steps because the PRISM estimates within our date range were only available on a monthly timescale. Because we used a hierarchical model, we could have sampled SPEI and SPI as parameters from the distributions specified in Vicente‐Serrano et al. (2010). However, we computed each set of SPEI and SPI values using the SPEI package in the R programming environment (Beguera and Vicente‐Serrano 2013) and then stored those values. We then sampled from that range of values using a model fitting algorithm described below.

### Statistical analyses

We modeled our response variable using a Bayesian representation of a hierarchical beta regression (Figueroa‐Zúñiga et al. 2013). While it may be possible to use a logistic regression to model continuous proportion data, typical logistic regression packages (e.g., in R) assume the data are binomially distributed (i.e., discrete and assume values of zero or one). The beta distribution, on the other hand, is a continuous distribution that assumes data are distributed on the interval [0,1] (Bolker 2008). However, the distribution is undefined at 0 or 1. For our data, digitization errors occasionally resulted in observations greater than 1.0 and we did observe a few zeros. For these cases, we recoded these observations as 0.99999 and 1 − 0.99999, respectively. These extreme observations made up less than 5% of our data, so we regarded this transformation as having minimal effect on our inferences. Had we made more 0 or 1 observations we would have used a zero‐inflated version of the beta likelihood (Ospina and Ferrari 2012).

We assumed the model for our observations was: $y_{ij}∼\beta \left(\mu_{ij}*\phi_{ij},\left(1-\mu_{ij}\right)*\;\;\phi_{ij}\right)$, where *μ* represented the mean and ϕ represented the dispersion parameter of the beta distribution, *i* represented each wetland and *j* represented each observation year for each wetland. Because this was a hierarchical model, we were able to model these two parameters as functions of another model. In this case, we modeled the mean with $\text{logit}\left(\mu_{ij}\right)=\beta_{\mu }X_{ij}+\gamma_{i}+\delta_{ij}$, where *β* represented a collection of regression parameters, *γ* represented a wetland‐specific random effect and *δ* represented residual error. We modeled the dispersion parameter with $\text{log}\left(\phi_{ij}\right)=\beta_{\phi }Z_{ij}+\varepsilon_{i}+\zeta_{ij}$, where **Z** was a vector of ones, *ε* was a wetland‐specific random effect and *ζ* represented residual error. We assumed that all random effect terms were normally distributed with a mean of zero and a variance term to be estimated. We parameterized the variance terms as precision terms (i.e., *τ* = 1/*σ*), which allowed us to model these terms assuming inverse‐gamma distributions. The model we used for *μ* assumed an intercept, a term for era (historical [pre‐1970] and contemporary [post‐2003]), terms for both SPEI and SPI, an interaction between era and SPEI, an interaction between SPEI and SPI and a three‐way interaction between SPEI, SPI, and era.

We considered the era term because we assumed the proportion area would be higher in the contemporary era, compared with the historical era. We considered the interaction between SPEI and SPI because we assumed that wetter periods in the short term (SPI) might have a larger effect during a drier period over the longer term (SPEI). We considered the interaction between SPEI and era because we expected that the response to long‐term climate would be different between eras. Lastly, we considered the three‐way interaction because we expected the short‐ and long‐term climate response to vary by era as well. Again, we assumed that the timescale *t* for the climate indices was also a parameter to be estimated conditional on all of the other model parameters. We assumed regular uninformative normal and gamma priors on regression parameters and an uninformative Dirichlet prior on *t*.

We fit this model using Markov Chain Monte Carlo (MCMC) simulation and wrote the algorithm in the R programming environment (R Core Team 2015). We used a combination of Gibbs sampling, which simulates draws from the full conditional posterior distribution (i.e., likelihood × prior) and Metropolis–Hastings (M‐H) updating, when the full conditional could not be calculated. In essence, the algorithm proceeded this way: (1) Use M‐H to propose new values for each $\mu_{i,j}$ or $\phi_{i,j}$ and accept or reject based on the posterior; (2) use Gibbs sampling to update all of the regression parameters; (3) use M‐H to propose new values of *t* for each era and index (1–120 for SPEI and 1–3 for SPI) and accept or reject based on the posterior; (4) go back to the step 1.

Models fit with this type of algorithm are typically summarized by computing statistics based on one or more Markov chains with different starting values. We ran three separate chains each with a 10,000 iteration burn‐in period to allow the algorithm to settle into a stationary sampling distribution, after which, we ran each chain for an additional 100,000 iterations. We then thinned each chain to every 100th iteration to reduce serial autocorrelation and computed Gelman–Rubin diagnostics to assure convergence (Gilks et al. 1995). We then combined the chains and summarized them by computing summary statistics for each posterior parameter distribution. We also diagnosed model fit by simulating data from the posterior parameter distributions and then regressing the simulated data against our actual observations. We did this assuming both “fixed effects" (marginal) and “random effects" (conditional). We report model fit statistics as the coefficients of determination ($R^{2}$) under both the marginal and conditional cases.

## Results

**Figure 1:**
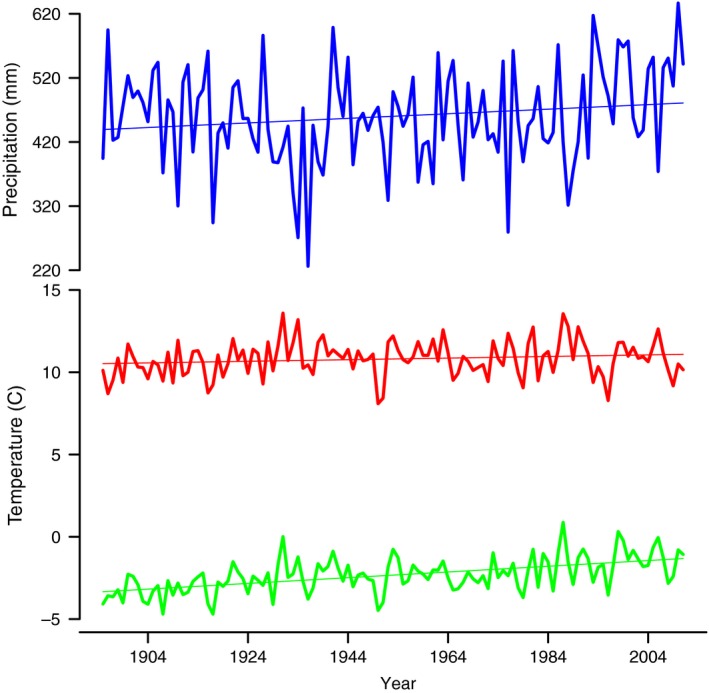
Summary of weather information for a set of sample wetlands in North Dakota. These summaries were generated from interpolated temperature and precipitation values from the Parameter‐elevation Regressions on Independent Slopes Model (PRISM). The top panel shows total annual precipitation in millimeters, while the bottom panel shows the annual mean of average monthly high temperature (red line) and average monthly low temperature (green line). Monthly values of these three measurements were used in calculating potential evapotranspiration.

The wetlands we analyzed in our study showed a general trend toward being less full in the historical era (mean = 0.34 ha, SD =  0.27) compared with the contemporary era (mean = 0.59 ha, SD = 0.27). Annual summaries of the temperature information used in computing our monthly climate indices did show a slight increasing average annual trend for both minimum temperature and for precipitation (Fig. 1). The parameter estimates from our model suggested that era and SPEI were useful variables in predicting the proportion of a basin's area that was covered with water, whereas SPI had a very weak and uncertain relationship with our response variable (Table 1). All further summaries and predictions were made assuming that SPI was held at its mean value. The mean estimate of the model's marginal predictive performance was $R_{\mathrm{marginal}}^{2}$ = 0.16. The model's conditional predictive performance showed some improvement ($R_{\mathrm{conditional}}^{2}$ = 0.61), which indicates additional sources of variation in the observed pattern for our response variable.

**Table 1:** Model parameter table showing posterior distribution summary statistics for a beta regression explaining variation in the proportion of a wetland basin covered with water in North Dakota, U.S.A. The distributions were summarized by their means, standard errors, and 95% quantiles. The *β* and ϕ terms represent regression parameters for mean (logit scale) and dispersion (log scale) parameters of a beta distribution, respectively. The *τ*s represent precision terms (i.e., inverse of the variance), and *t*s represent the timescale parameter for computing two indices: Standardized Precipitation Evapotranspiration Index (SPEI) and the Standardized Precipitation Index (SPI)

	Mean	SE	2.5%	97.5%
$\beta_{0}$	−0.62	0.13	−0.89	−0.38
$\beta_{\mathrm{era}}$	0.65	0.12	0.38	0.85
$\beta_{\mathrm{SPEI}}$	0.68	0.06	0.56	0.79
$\beta_{\mathrm{SPI}}$	−0.02	0.03	−0.08	0.04
$\beta_{\mathrm{SPEI}\;\;*\;\;\mathrm{era}}$	−0.32	0.08	−0.48	−0.14
$\beta_{\mathrm{SPEI}\;\;*\;\;\mathrm{SPI}}$	−0.00	0.04	−0.09	0.08
$\beta_{\mathrm{SPEI}\;\;*\;\;\mathrm{SPI}\;\;*\;\;\mathrm{period}}$	0.00	0.05	−0.10	0.10
$\tau_{\gamma }$	19.70	4.51	12.92	30.45
$\tau_{\delta }$	0.51	0.06	0.39	0.64
log(ϕ)	3.65	0.14	3.38	3.95
$\tau_{\varepsilon }$	0.20	0.02	0.16	0.24
$\tau_{\zeta }$	0.51	0.06	0.39	0.64
$t_{{\mathrm{SPEI}}_{\mathrm{historical}}}$	83.75	18.24	49.00	118.00
$t_{{\mathrm{SPEI}}_{\mathrm{contemporary}}}$	63.96	15.27	39.00	92.00
$t_{\mathrm{SPI}}$	2.03	0.85	1.00	3.00

**Figure 2:**
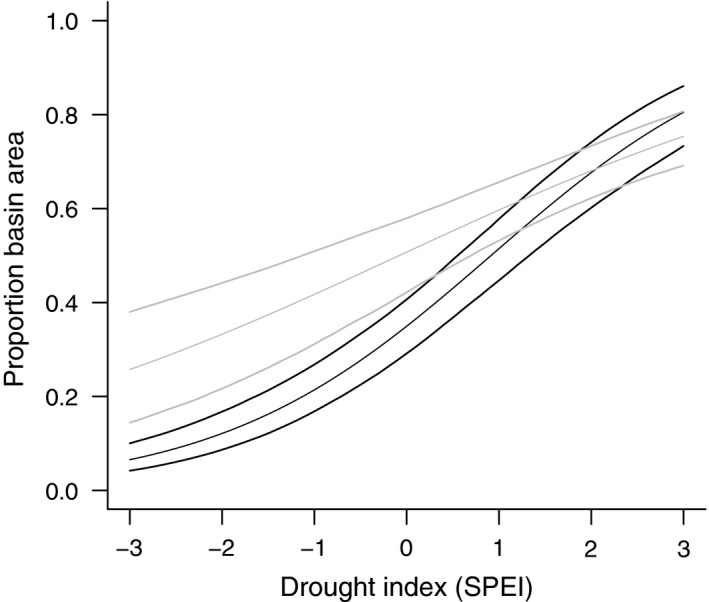
Predicted relationship between the observed proportion of a wetland basin that was covered with water and Standardized Precipitation Evapotranspiration Index in North Dakota, U.S.A. The thin black line represents the posterior mean relationship for observations made prior to 1970 and the thicker lines represent 95% Bayesian credible intervals. The gray lines represent the average relationship for observations made after 2003.

Examining the parameter estimates showed that the proportion of a wetland's area that was covered with water positively varied with SPEI, suggesting that drier periods resulted in lower proportions and wetter periods resulted in higher proportions. Likewise, wetlands in the contemporary era appeared to have a larger proportion of their basin areas covered with water compared to the historical era. The degree of change in these proportions in response to changes in SPEI also appeared to vary between the two eras. Wetlands tended to be more full, but less responsive to SPEI during the post‐2003 then the pre‐1970 era (Fig. 2).

**Figure 3:**
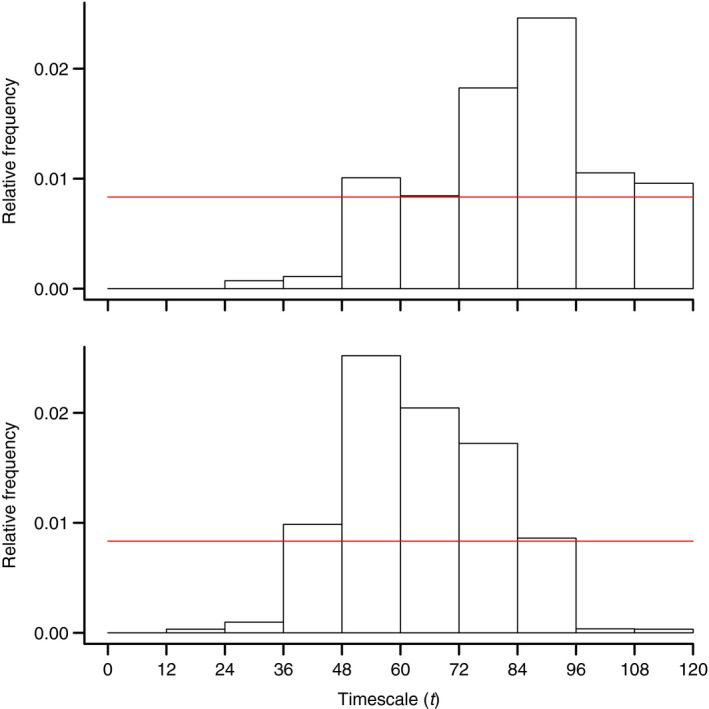
Posterior distributions for the temporal scaling parameter *t*, used in computing the relationship between the proportion of a wetland basin that was covered with water and the Standardized Precipitation Evapotranspiration Index. The top panel represents the estimated distribution for the historical period (i.e., pre‐1970) and the lower panel represents the estimated distribution for the contemporary period (post‐2003). The height of the bars represents the relative frequency with which certain values of *t* were chosen in our analysis. The thin red line represents the expected frequency under a uniform prior distribution. The parameter *t* was modeled as a discrete distribution in monthly increments and could assume values between 1 and 120 months. Frequencies were assigned to one of 10 annual bins to smooth the distributions for presentation.

On average, the scaling of SPEI also varied between the two eras (Table 1), although there was considerable uncertainty around the mean of each distribution. Despite this, there appeared to be enough information in the data to estimate a posterior distribution that was considerably different than the uniform prior that we specified (Fig. 3). The distributional plots also showed clear shifts in the modes of each distribution, which indicated that a shorter timescale fit the data better in the contemporary era compared with the historical era.

**Figure 4:**
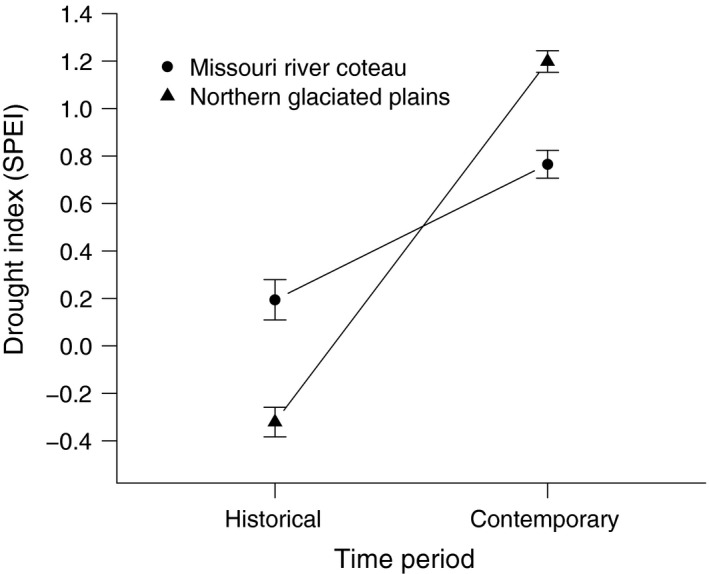
Plot of average Standardized Precipitation Evapotranspiration Index values (±95% BCI) computed for sample wetlands in two different regions of North Dakota (Missouri River Coteau and Northern Glaciated Plains) in a historical era (pre‐1970) and a contemporary era (post‐2003).

**Figure 5:**
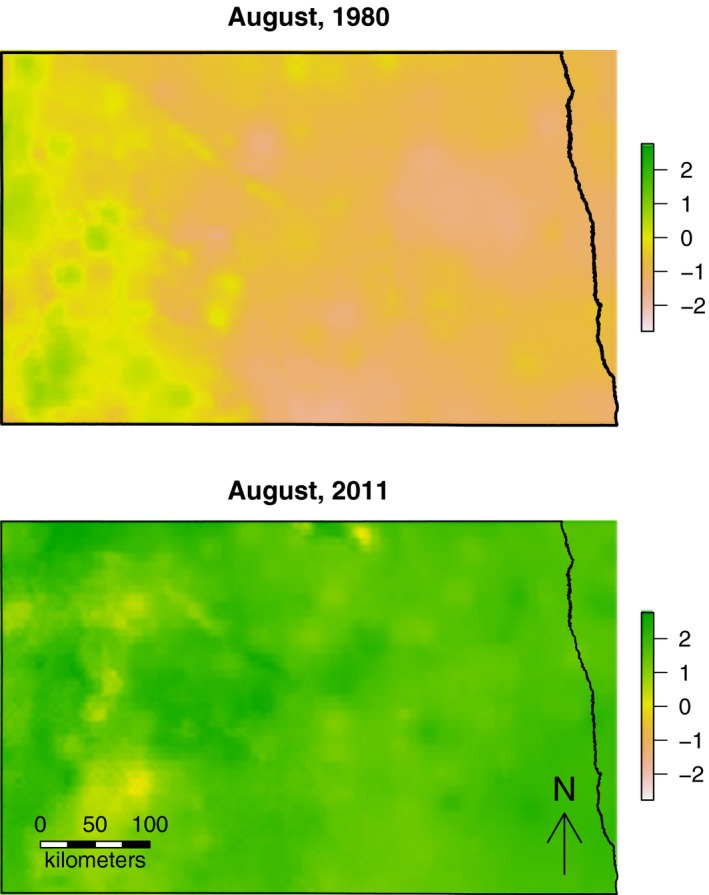
Example of weighted Standardized Precipitation Evapo‐transpiration Index (SPEI) values for North Dakota, U.S.A. (black outline) for the month of August in the year 1980 (top) and 2011 (bottom). The SPEI values were computed using parameter‐elevation regressions on independent slopes model output and a distribution of timescale values (see Fig. 3).

One potential reason for the effect of era on both the estimated response to climate and the shift in *t* could be that the historical era was drier than the contemporary era. To analyze this, we computed a weighted climate index, which was simply the sum of each possible index weighted by the likelihood of that index from our estimated distribution. We then regressed that index according to the era and the region which corresponded to that index. The results of this post hoc analysis suggested that the west‐central part of North Dakota (Missouri River Coteau region) shifted from being drier to being wetter, whereas the eastern part of the state (Northern Glaciated Plains region) showed a similar, but more exaggerated, pattern (Fig. 4). Interestingly, we did try fitting a region parameter in our beta regression model, but those estimates did not explain any variation in the data. Spatial plots of our weighted index for the month of August, for example, confirmed the regional wetting pattern, but also showed that the index was more spatially variable historically (Fig. 5).

**Figure 6:**
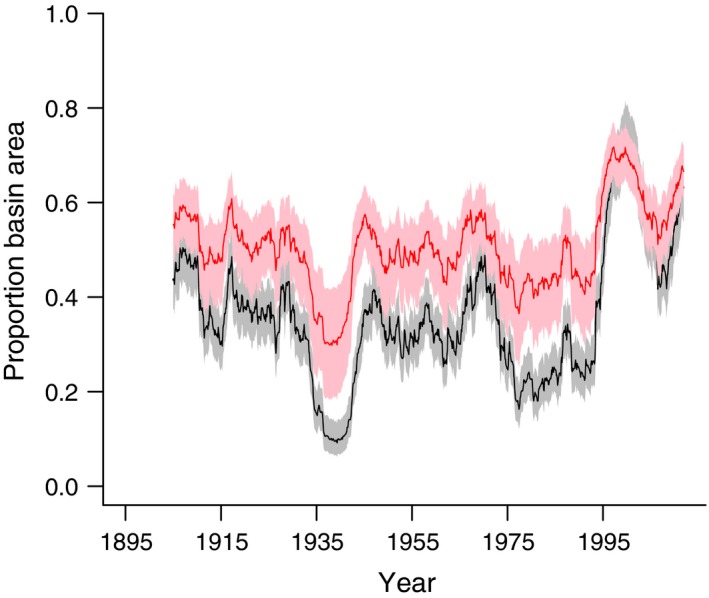
Predicted water levels over time for a single wetland in North Dakota, U.S.A. The black line represents average predictions of the proportion of the basin covered with water assuming conditions prior to 1970 surrounded by 95% Bayesian credible intervals (gray). The red line represents average predictions assuming conditions after 2003 surrounded by 95% Bayesian credible intervals (pink). The predictions were made based on Standardized Precipitation Evapotranspiration Index values computed for each year assuming a distribution of possible values for timescale (see Fig. 3).

Using this same weighted climate index, we predicted the expected proportion of a basin to be covered with water using one of the wetlands from our sample as an example (Fig. 6). In general, the predicted patterns suggested that the amount of water covering a wetland historically tended to respond much more strongly to both dry and wet periods. Under more contemporary conditions, there were somewhat similar relative fluctuations in water surface area, but overall wetlands were much fuller, and responded over slightly shorter time periods compared with historical dynamics.

## Discussion

Our work suggests that differences in climate may be responsible for some, but not all, of the dynamics we observed in the amount of water that wetland basins hold. The relationship between water areas and relative wetness and dryness was very similar to those already observed and reported in McCauley et al. (2015). Their work used a constant timescale parameter of ten years for SPEI in their model of water surface areas, but they did report that when conducting exploratory analyses shorter timescales tended to better explain more contemporary observations. However, they suggested that the range of values computed for drought indices were similar, and thus, climates between periods were similar. Our analysis, on the other hand, formalized the estimation of a change in climate scaling, rather than selecting this parameter in a step‐wise fashion. As a result, we were able to estimate the degree of shift in the scaling parameter and draw inference from that parameter. What our results suggest is that it now takes a slightly shorter period of accumulation of wet and dry periods over time to affect the hydroperiods of wetlands compared to the past. The fact that it may take multiple years to affect a change in water conditions for wetlands with long hydroperiods is not surprising given that other work shows that water conditions tend to be correlated with conditions in previous years (Niemuth et al. 2010; Huang et al. 2011). This suggests that moisture deficits accumulated over years might be more useful in predicting surface areas in wetlands within more closed basins. But there may also be more inference to be made based on the scaling parameter. One way to interpret the timescale used in the calculation of SPEI may be the degree to which surface inputs or connection to groundwater explain variation in hydroperiods (e.g., Skøien et al. 2003).

Others have pointed out that groundwater interactions with permanent and semipermanent wetlands are complex, but that such groundwater interaction could attenuate the influence of dry periods (LaBaugh et al. 1996, 1998). In addition, deeper groundwater in this region has been reported to move as slowly 0.0006 m year${}^{-1}$ (Sloan 1972), but shallower groundwater might move much faster (e.g., 1000 m year${}^{-1}$ van der Kamp and Hayashi 2009). Given the change in timescale between the historical and the contemporary observations, our results would suggest that our wetlands have become slightly more influenced by surface inputs, such as drainage water. This finding is consistent with the expectation that more drainage of wetlands in a landscape could lead to more consolidated wetlands that are larger and less responsive to wetting or drying periods (Anteau 2012; McCauley et al. 2015).

On the other hand, our results may suggest that the relative change in the amount of water a wetland holds under dry or wet conditions may be because of changes in climate variability. We suggest that the scaling parameter is a potentially useful indicator of the degree of variation in wet and dry periods. In other words, as the timescale parameter decreases the degree of variability represented by the index also generally increases. Larger values of the timescale parameter tend to average over much of this variability. A change in this parameter could, thus, represent a shift in the periodicity of fluctuations between wet and dry periods over the longer term. Our finding that average index values have shifted between eras and regions may indicate that perhaps the differences in climate between eras might be responsible for the variability we observed in water surface areas. That is, the PPR portion of North Dakota may be experiencing wetter periods, which may raise water tables in this region. Raised water tables could result in wetlands having more groundwater connection, which could increase and stabilize water surface areas. However, one cannot rule out the fact that our results could have been caused by the asymmetric distribution of our observations between eras. The observations that McCauley et al. (2015) made in the historical period spanned about 32 years, whereas the observations in the contemporary period only spanned 7 years. Thus, the data for the contemporary period may not span enough time to determine whether there was an actual change in average climate. The relative strength of our results suggests that there were additional sources of variation in wetland water levels. One potentially strong source was found by McCauley et al. (2015), who were able to link increased water surface areas and attenuated responses to SPEI to land use parameters. These findings support that idea of increased wetland drainage and increased surface inputs causing some of the changes in water level responses between eras.

Despite not conclusively being able to address the question of climate shifts, the Bayesian nature of our analysis would allow us to improve our inferences over time. We could, for example, treat our current parameter estimates and predictions as priors for further analysis. This means that we could collect more data in the future from other wetlands within North Dakota and use these data to update our estimates over time. Such an approach would have the advantage of more closely mirroring how we as scientists learn from data and would also improve the precision and accuracy of estimates through time. One would also not have to completely reanalyze an entire dataset, but could instead focus on how much information new data actually contain. The analytical framework we developed here also allowed us to improve upon previous studies using this index (e.g., Vicente‐Serrano et al. 2012, 2013) by treating the timescale as an unobservable parameter to be estimated. We contend that our approach is more informative than using variable selection procedures or simply choosing a value in an ad hoc fashion, because such approaches do not make distributional assumptions, and subsequently do not allow for the inclusion of prior information and learning. We suggest that further improvements should include modeling timescale as a temporally continuous parameter, so one can identify when climate changes are likely occurring based on relationships found in data.

One final benefit we noted in using our approach was that it essentially allowed the amount of temporal autocorrelation in the index to be estimated relative to its explanatory value given the data. For the system we analyzed in this study, we expected a potentially high degree of autocorrelation would help explain variation in wetland water surface areas. But this may not be the case for other systems. For instance, those systems that respond to more seasonal climate phenomena might require shorter nonoverlapping timescales. One could accomplish this by simply setting different prior assumptions on the distribution of timescale values and then estimating the posterior distribution for timescales, as we did in our analysis. We suggest that modeling approaches, like the one we present here, along with the spatial and temporal flexibility that SPEI offers, be considered as part of the toolbox for understanding how ecological systems respond to climate.

## Confict of Interest

None declared.
